# Individual and Combined Effects of Diseases and Cytological Endometritis on Reproductive Performance and Culling of Dairy Cows: Preliminary Results

**DOI:** 10.3390/ani12212913

**Published:** 2022-10-24

**Authors:** Merle Valdmann, Jevgeni Kurykin, Andres Waldmann

**Affiliations:** 1 Institute of Veterinary Medicine and Animal Sciences, Estonian University of Life Sciences, 51006 Tartu, Estonia; 2 Faculty of Veterinary Medicine, Latvia University of Life Sciences and Technologies, LV-3004 Jelgava, Latvia

**Keywords:** disease, cytological endometritis, onset of luteal activity, reproductive performance, culling, dairy cow, far-reaching consequences

## Abstract

**Simple Summary:**

Dairy cow longevity is a major concern in most high milk-producing countries. Failure to reproduce is the most common reason for involuntary culling worldwide. A high prevalence of metabolic and infectious diseases (DIS) in dairy herds is not unusual; however, the effects of DIS on cow fertility vary and the reason for this variation is currently unclear. One and a half months after parturition, approximately 30% of cows experience uterine inflammation characterised by an increased proportion of white blood cells, more specifically polymorphonuclear neutrophils in the uterine lumen—a disease termed cytological endometritis (CYTO). CYTO is associated with a decreased conception rate and higher culling risk. Accordingly, we studied the influence of CYTO on the fertility and culling of clinically healthy and unhealthy dairy cows. We found that CYTO+ status, irrespective of DIS status, decreased the pregnancy rate. When DIS+ and CYTO+ were combined, they had an additive negative effect. This finding led us to conclude that the variation in the effects of clinical disease on reproduction can at least partly be explained by the occurrence of CYTO. Therefore, in dairy herds with fertility problems, the prevalence of CYTO should be assessed. Further investigations including a larger number of cows are required to confirm these results.

**Abstract:**

The objective of this study was to evaluate the effect of calving-related disorders/clinical diseases (DIS) and cytological endometritis (CYTO) on the reproductive performance and culling of dairy cows. In a total of 119 multiparous Holstein cows, DIS were recorded and CYTO was diagnosed at 40 ± 2 d in milk. Onset of luteal activity was defined as the first postpartum measurement of milk progesterone >5 ng/mL. A dummy variable containing all four possible permutations between DIS and CYTO statuses was created. The pregnancy rates after first artificial insemination were 57.7, 42.9, 23.0 and 15.8% for the DIS−/CYTO−, DIS+/CYTO−, DIS−/CYTO+ and DIS+/CYTO+ groups, respectively. The hazard of pregnancy was affected by DIS−/CYTO+ and DIS+/CYTO+ health statuses (hazard ratio (HR) = 0.43 and 0.29, respectively), whereas DIS+/CYTO− and DIS−/CYTO− cows had a similar hazard to pregnancy. The hazard of onset of luteal activity was affected by DIS+/CYTO+ health status (HR = 0.45), but not by DIS+/CYTO− and DIS−/CYTO+ health statuses. Compared with DIS−/CYTO− cows, DIS−/CYTO+ and DIS+/CYTO+ cows had 4.24 and 5.57 times the odds of being culled, respectively. Culling was not affected by DIS+/CYTO− health status. In conclusion, CYTO+ status, irrespective of DIS status, decreased the pregnancy rate. When DIS+ and CYTO+ were combined, they had an additive negative effect. Our preliminary findings suggest that the far-reaching consequences of clinical diseases on fertility and culling can be mediated through the development of CYTO. Further investigations including a larger number of cows are required to confirm these results.

## 1. Introduction

Dairy cow longevity has decreased over the years in most high milk-producing countries, with failure to reproduce being the most common reason for involuntary culling worldwide [1]. Poor reproductive performance and curtailed longevity of dairy cows limit the achievements of a sustainable dairy industry, increase the environmental footprint of milk production and expose animal welfare issues.

During the periparturient transition period, dairy cows are subjected to a variety of metabolic and physiological changes that affect productivity, health and future reproductive performance. Despite tight homeostatic controls and homeorhetic adjustments to cope with the changes in metabolism, a high prevalence of calving-related disorders and metabolic or infectious diseases is not uncommon, with 45–71% of dairy cows across different levels of milk production, breeds and management systems affected [2,3,4,5,6,7]. Compromised postpartum health has a negative effect on the performance and survival of dairy cows. However, having had a disease does not necessarily put a cow at greater risk of being culled, unless she is unable to become pregnant [8].

Fourichon et al. [9] with data from 70 studies found that the estimates of disease effects on fertility measures were often heterogeneous between studies; however, the authors could not fully explain the source of this variance [9].

Cytological endometritis (CYTO) is a postpartum uterine inflammation characterised by an increased percentage of inflammatory cells, principally polymorphonuclear neutrophils (PMN) in endometrial cytology samples, which results in a significant reduction in reproductive performance [10,11]. CYTO effects on average 30% of cows at 30 to 60 days postpartum, with the prevalence ranging from 5–80% in some herds [12,13,14,15]. As approximately 75% of all clinical diseases in dairy cows are diagnosed in the first 3 to 4 weeks of lactation [4,16], the probability of a calving-related disorder or clinical disease preceding the diagnosis of CYTO is high. It is thought that the development of CYTO is a consequence of a lack of energy, metabolic stress and systemic inflammation leading to impaired uterine immune function [12,17,18,19].

At present, there are no data on the combined effects of calving-related disorders/clinical diseases and CYTO on the reproductive performance and culling of dairy cows. Previous investigations [4,6,9,20,21,22,23] studying the effects of health-related events and diseases on reproduction did not account for the possible confounding effects of CYTO.

The present study is the first of its kind in that it evaluates the individual and combined effects of diseases and CYTO on the reproductive performance and culling of dairy cows. Therefore, the central hypothesis of this study is that the variance in the effect of clinical diseases on reproduction and culling is related to the CYTO status of the animals. Specifically, unhealthy cows with a CYTO-positive diagnosis resume postpartum luteal activity later, suffer greater detrimental effects concerning fertility and have a higher culling risk compared to unhealthy cows with CYTO-negative health status.

Because an increase in parity is associated with inferior reproductive performance [24,25,26] and higher culling rate [27], and environment can affect pregnancy outcome, our study sample was comprised of only multiparous Holstein cows kept under identical environmental conditions. We evaluated the individual and combined effects of disease and CYTO status on the onset of luteal activity, pregnancy rate after first artificial insemination (AI), time to pregnancy, culling risk and first 45-day cumulative milk yield.

The preliminary findings presented here suggest that the far-reaching consequences of clinical diseases in early postpartum on future fertility and culling can be mediated through the development of CYTO.

## 2. Materials and Methods

### 2.1. Animals

This was a retrospective cohort study. A description of the studied animals has previously been reported [15]. Briefly, 119 multiparous Holstein cows from a single 1200-cow free-stall commercial dairy herd with 305 d average milk yield of >8500 kg were used. Experimental cows formed three consecutive series of calvings. Cows were enrolled three weeks before their expected calving date and were followed to at least day 65 of pregnancy or culling from the herd. Cows were fed their respective diets as total mixed ration offered twice daily for *ad libitum* intake. Cows were milked twice daily with parallel rotary milking parlour (PER-40, DeLaval, Tumba, Sweden) and daily milk yields were measured with flow milk meters (MM25, DeLaval, Tumba, Sweden). The cows were selected for AI on spontaneous oestrus, detected by collar mounted activity meters (ALPRO, DeLaval, Tumba, Sweden) combined with visual observation for standing to be mounted and secondary oestrous signs. Cows were inseminated with frozen-thawed semen of proven Holstein sires after a voluntary waiting period of 60 days. One experienced herd AI technician conducted all the inseminations. The AI technician was blinded to cow history and health status. Pregnancies were diagnosed from milk progesterone (P4) profiles and confirmed by rectal palpation at 65 to 70 days after AI. The cows were vaccinated against bovine viral diarrhea and infectious bovine rhinotracheitis viruses.

### 2.2. Evaluation of Health Status

Calving-related conditions and clinical diseases were diagnosed, confirmed, documented and treated by an experienced farm veterinarian. There was no indication of presence of infectious diseases. The conditions and diseases diagnosed included twinning, dystocia, retained placenta, metritis, clinical endometritis, clinical hypocalcaemia, ketosis, clinical mastitis and severe lameness. Dystocia was defined as providing assistance without or with the use of mechanical traction. Retention of foetal membranes was characterised as the presence of foetal membranes at least 12 h after calving. Metritis (watery red-brown fluid to viscous off-white purulent uterine discharge independent of fever before 21 days in milk (DIM)) and clinical endometritis (purulent vaginal discharge after 21 DIM) were defined according to Sheldon et al. [28]. Clinical hypocalcaemia was identified from the resolution of hypocalcaemia symptoms (incoordination when walking, decrease in body temperature and downer cow syndrome) after infusion of 10 g of calcium as calcium borogluconate. Ketosis was determined from reduced feed intake and/or milk yield and milk testing positive for ketone bodies (Ketostix strip; Bayer, Leverkusen, Germany). Clinical mastitis was characterised as the presence of abnormal milk or signs of inflammation in one or more quarters. All cows were examined for signs of clinical mastitis before each milking.

### 2.3. Endometrial Sampling, Diagnostic Criterion for Cytological Endometritis

Endometrial samples for cytological examination were collected at 40 ± 2 (mean ± SD) days after calving using an endometrial brush (Uterobrush^®^; Medscand Medical, Malmö, Sweden) attached to a stainless-steel device for use in cows (European Patent EP2029026B1). Before use, the devices were mounted with Uterobrush^®^, individually packed in 75-mm Steriking^®^ transparent heat-sealable roll (Wipak Oy, Nastola, Finland) and sterilised by autoclaving. After thorough cleaning of the vulva, endometrial cytology samples were taken from the base of the larger uterine horn. Under guidance per rectum, the device was introduced into the desired region of the uterus, the rod-fixing screw was loosened and the rod of the device was pushed into the guiding tube. As a result, the Uterobrush^®^ advanced into the uterus. By rotating the rod multiple times around its axle, endometrium material was collected on the Uterobrush^®^. The Uterobrush^®^ was then retracted into the device, the rod was fixed with the aid of the rod-fixing screw and the device was taken out of the uterus. Between uses, the devices were washed, equipped with new Uterobrush^®^, individually packed and autoclaved. All cytology samples were taken by the same researcher. Slides for cytologic examination were prepared by rolling the Uterobrush^®^ onto a clean glass microscope slide and immediately fixing in a current of warm air using a blow drier. Four slides from each cow were prepared. The slides were then transferred to the laboratory and two of the four slides from each cow were stained with May-Grünwald Giemsa procedure and examined by light microscopy under 400× and 1000× magnification (Olympus BX51; Olympus, Tokyo, Japan). For each slide, surveying 100 cells, the numbers of epithelial cells and PMN were counted and the percentage of PMN calculated as described by Kasimanickam et al. [10]. The average percentage of PMN of the two slides was used for analyses. All slides were evaluated by the same cytologist who had no knowledge of the health status of the animals. The diagnostic criterion for CYTO was set at a PMN value giving the greatest summation of sensitivity and specificity [12] in predicting non-pregnancy by 120 and 180 days postpartum. The optimum cut-off was greater than 8% PMN with sensitivities (95% CI) 44.7 (31.2–57.6) and 53.9 (37.2–69.9), and specificities 83.3 (71.5–91.7) and 81.3% (71.0–89.1) for the 120- and 180-day time-points, respectively. The farm veterinarian, farm manager and AI technician were unaware of the cytology test results.

### 2.4. Milk Sample Collection and Milk Progesterone Analysis

Milk samples were taken twice weekly for P4 measurement until confirmation of pregnancy by rectal palpation or culling from the herd. In order to negate the effect of time of milk extraction on P4 concentration, samples were collected within 10 min following PM milking in the milking parlour [29]. A volume of 10–15 mL milk was withdrawn by stripping and collected in plastic tubes containing potassium dichromate for preservation. Samples were frozen at –20 °C until P4 analysis. Concentrations of P4 in milk were measured by an enzyme immunoassay [30], which was modified by using the second antibody technique. The specificities of the monoclonal antibody and assay have previously been reported [31]. The inter-assay and intra-assay coefficients of variation were <10%. The limit of sensitivity using a 20 µL sample was <0.5 ng/mL.

### 2.5. Onset of Luteal Activity Definition

Onset of luteal activity was defined as the first postpartum measurement of milk progesterone >5 ng/mL [30,32].

### 2.6. Body Condition Scoring

Cows were assessed for body condition score (BCS) at week +3 postpartum using a 1 (emaciated) to 5 (obese) scale with 0.25-unit increments according to Ferguson et al. [33]. Cows with BCS ≤ 2.5 were classified as low BCS and those with BCS ≥ 2.75 to 3.75 as moderate BCS.

### 2.7. Locomotion Scoring

Locomotion was scored every fortnight on a 5-point scale in increments of 1 using the method described by Sprecher et al. [34]. Cows with a locomotion score of at least 4 were classified as severely lame.

### 2.8. Statistical Analysis

To evaluate the individual and combined effects of calving-related conditions/clinical diseases (DIS) and CYTO, a dummy variable containing all four possible permutations between health and CYTO statuses was created: condition/disease-negative—CYTO−negative (DIS−/CYTO−); condition/disease-positive—CYTO-negative (DIS+/CYTO−); condition/disease-negative—CYTO-positive (DIS−/CYTO+); and condition/disease-positive—CYTO-positive (DIS+/CYTO+).

Parity was created as a binary variable including lactation number 2 and >2.

To determine the distribution of different clinical diseases between DIS+/CYTO+ and DIS+/CYTO− cows, the DIS+ cows were grouped into three categories as follows: (1) cows that had clinical uterine inflammatory disease only, which included purulent vaginal discharge (metritis/clinical endometritis); (2) cows that had clinical uterine inflammatory disease and were also affected by another calving-related condition/disease; and (3) cows that had only non-uterine diseases, which included clinical mastitis, clinical hypocalcaemia and severe lameness.

The effects of health and CYTO statuses (DIS/CYTO group) on pregnancy to first service and culling were analysed by logistic regression. The effects of DIS/CYTO group on onset of luteal activity and time to pregnancy were determined by Cox’s proportional hazard models. The survival time was right censored at 255 DIM or at the date of culling if culling occurred before 255 DIM. The proportional hazards assumption was tested in R using statistical software R 3.3.3 (R Foundation for Statistical Computing, Vienna, Austria) using cox.zph in the survival package [35].

First 45-day cumulative milk yields among the four DIS/CYTO groups were compared by ANCOVA. *p*-values were Bonferroni corrected. Shapiro-Wilk test was used to analyse the distribution of residuals.

The potential confounding effects of parity (2nd, >2nd), BCS at week +3 postpartum (BCS ≤ 2.5, BCS ≥ 2.75) and time from calving to first AI were tested univariately and, if associated with outcome (*p* < 0.2), submitted to the multivariable model(s). The covariates were retained in the multivariable model(s) if associated with outcome (*p* < 0.15).

Proportions were compared with Chi-square test. Statistical significance was set at *p* < 0.05 and trend being between ***p*** ≥ 0.05 and *p* < 0.10.

MedCalc^®^ Statistical Software version 20.109 (MedCalc Software Ltd., Ostend, Belgium) was used for statistical analyses and preparation of survival curves. Figures were prepared using Prism 8 for Windows V.8.4.3 (GraphPad Software, San Diego, CA, USA).

## 3. Results

### 3.1. Description of the Studied Cows

Descriptive statistics of the measured parameters in the studied cows are given in Table 1.

The proportions of cows affected by twinning, retained placenta, metritis/purulent vaginal discharge, clinical mastitis, clinical hypocalcaemia, severe lameness and CYTO were 6.7, 5.9, 30.3, 7.6, 1.7, 5.9 and 30.3%, respectively. In the 119 studied cows, 20 (16.8%) had more than one health disorder/problem and 68 (57.1%) did not have any calving complication or health disorder/problem. The proportions of DIS−/CYTO−, DIS+/CYTO−, DIS−/CYTO+ and DIS+/CYTO+ cows were 45.4 (54/119), 24.4 (29/119), 11.8 (14/119) and 18.5% (22/119), respectively.

### 3.2. Disease and CYTO Effects on Pregnancy after First AI and Culling

**Figure 1:**
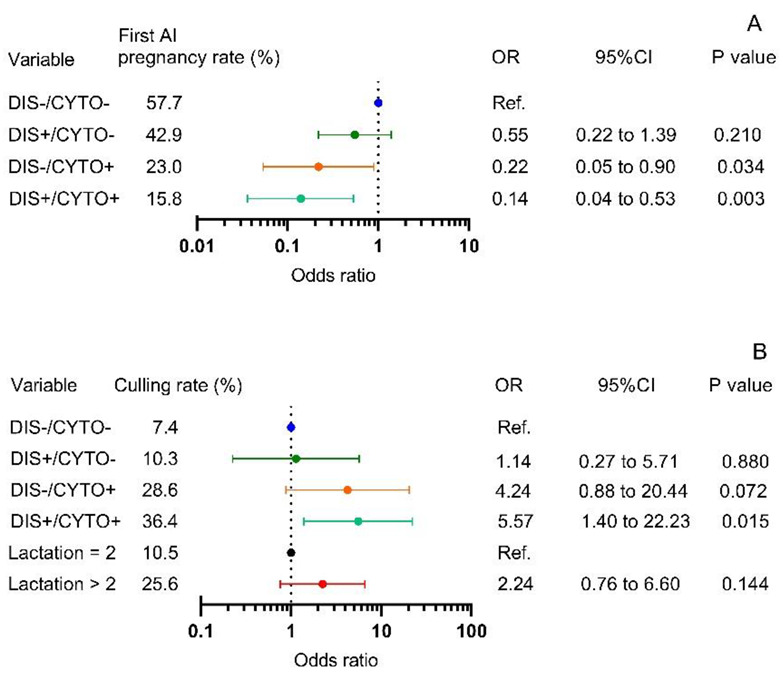
Odds ratios (OR) and 95% confidence intervals (CI) from logistic regression models for first artificial insemination (AI) pregnancy rate (*n* = 112) (**A**) and culling rate (*n* = 119) (**B**) for multiparous Holstein cows stratified by disease (DIS) and cytological endometritis (CYTO) statuses (DIS−/CYTO− = disease-negative/CYTO-negative; DIS+/CYTO− = disease-positive/CYTO-negative; DIS−/CYTO+ = disease-negative/CYTO-positive; DIS+/CYTO+ = disease-positive/CYTO-positive). OR for culling was adjusted for lactation number (=2 and >2). OR < 1 indicates a reduced relative risk; OR >1 indicates an increased relative risk.

DIS/CYTO group affected pregnancy after first AI (*p* = 0.004) and culling (*p* = 0.010). The proportions of cows pregnant after first AI were 57.7, 42.9, 23.0 and 15.8% and the proportions of cows culled were 7.4, 10.3, 28.6 and 36.4% for DIS−/CYTO−, DIS+/CYTO−, DIS−/CYTO+ and DIS+/CYTO+ cows, respectively. The logistic regression model (Figure 1) revealed that compared with healthy cows, DIS+/CYTO− status had a weaker negative effect on the first AI pregnancy rate (odds ratio (OR) = 0.55, *p* = 0.210) than DIS−/CYTO+ (OR = 0.22, *p* = 0.034) and DIS+/CYTO+ cows (OR = 0.14, *p* = 0.003). Accounting for the effect of parity (*p* = 0.14), the odds of culling in DIS+/CYTO− cows did not differ from that in DIS−/CYTO− cows (OR = 1.14, *p* = 0.880). DIS−/CYTO+ cows had 4.24 times the odds (*p* = 0.072) and DIS+/CYTO+ cows had 5.57 times the odds (*p* = 0.015) of being culled compared with DIS−/CYTO− cows.

### 3.3. Disease and CYTO Effects on Onset of Luteal Activity and Pregnancy

**Figure 2:**
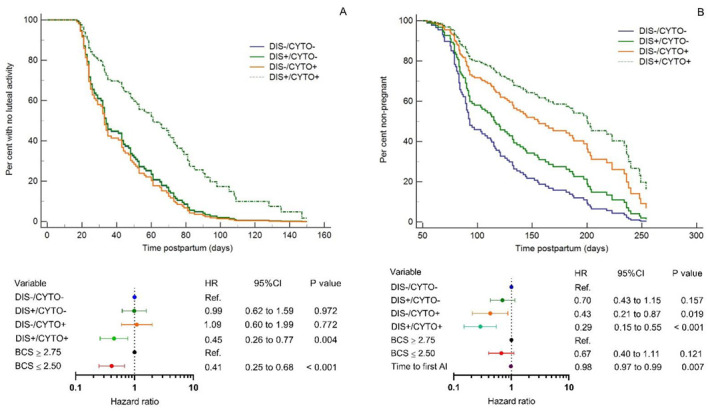
Multivariate Cox’s proportional hazard curves and adjusted hazard ratios (HR) with 95% confidence intervals (CI) for time to onset of luteal activity (*n* = 119) (**A**) and time to pregnancy (*n* = 112) (**B**) for multiparous Holstein cows stratified by disease (DIS) and cytological endometritis (CYTO) statuses (DIS−/CYTO− = disease-negative/CYTO-negative; DIS+/CYTO− = disease-positive/CYTO-negative; DIS−/CYTO+ = disease-negative/CYTO-positive; DIS+/CYTO+ = disease-positive/CYTO-positive). Time to onset of luteal activity was adjusted for body condition score (BCS) at week +3 postpartum (≥2.75 and ≤2.5). Onset of postpartum luteal activity was defined as the first milk progesterone rise above 5 ng/mL. Time to pregnancy was adjusted for time to first artificial insemination (AI) and BCS at week +3 postpartum (≥2.75 and ≤2.5). HR < 1 indicates a reduced daily relative risk; HR > 1 indicates an increased daily relative risk.

Accounting for BCS at week +3 postpartum effects (*p* < 0.001), DIS+/CYTO+ cows took a longer time to resume cyclicity (hazard ratio (HR) = 0.45, *p* = 0.004), whereas DIS+/CYTO− cows and DIS−/CYTO+ cows had a similar hazard to resume cyclicity (HR = 0.99, *p* = 0.972) and HR = 1.09, *p* = 0.772, respectively) when compared to DIS−/CYTO− cows (Figure 2). The interaction term BCS by health group was not statistically significant (*p* = 0.58) and was not incorporated into the model.

Accounting for BCS at week +3 postpartum (*p* = 0.121) and time to first AI (*p* = 0.007) effects, DIS−/CYTO+ and DIS+/CYTO+ cows took a longer time to pregnancy (HR = 0.43, *p* = 0.019 and HR = 0.29, *p* < 0.001, respectively), whereas DIS+/CYTO− cows had a similar hazard to pregnancy (HR = 0.70, *p* = 0.157) when compared to DIS−/CYTO− cows (Figure 2). The interaction terms BCS by health group and time to first AI by health group were not statistically significant with *p*-values of 0.75 and 0.42, respectively, and were not incorporated into the model.

### 3.4. Additive Effect of Clinical Disease and CYTO on Onset of Luteal Activity, Pregnancy and Culling

CYTO was a clinical disease effect modifier. The logistic regression models revealed that unhealthy cows with a negative CYTO diagnosis had superior fertility and lower culling risk when compared to unhealthy cows with a positive CYTO diagnosis. Specifically, DIS+/CYTO+ cows had 0.25 times the odds (95% CI = 0.06–1.06, *p* = 0.060) of being pregnant at first service when compared to DIS+/CYTO− cows. After adjusting for lactation number (=2 and >2), DIS+/CYTO+ cows had 4.91 times the odds (95% CI = 1.10–21.86, *p* = 0.037) of being culled when compared to DIS+/CYTO− cows.

After adjusting for BCS at week +3 postpartum (≥2.75 and ≤2.5), the Cox’s proportional hazard model showed that DIS+/CYTO+ cows were 55% less likely to resume cyclicity (HR = 0.45, 95% CI = 0.25–0.82, *p* = 0.009) when compared to DIS+/CYTO− cows. After adjusting for time to first AI and BCS at week +3 postpartum (≥2.75 and ≤2.5), the Cox’s proportional hazard model showed that DIS+/CYTO+ cows were 59% less likely to become pregnant (HR = 0.41, 95% CI = 0.21–0.81, *p* = 0.010) when compared to DIS+/CYTO− cows.

### 3.5. Distribution of Clinical Diseases between Unhealthy CYTO-Positive and Unhealthy CYTO-Negative Cows

**Figure 3:**
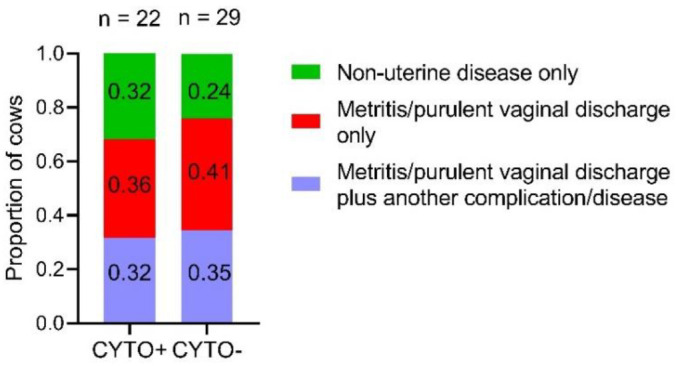
Proportions of cows with non-uterine disease only, metritis/purulent vaginal discharge only and metritis/purulent vaginal discharge plus another complication/disease between unhealthy cytological endometritis-positive (CYTO+) and unhealthy cytological endometritis-negative (CYTO−) multiparous Holstein cows. Out of the 119 studied cows, 51 (43%) had postpartum complications and/or disease.

The proportions of cows with non-uterine disease only, cows with metritis/purulent vaginal discharge only and cows with metritis/purulent vaginal discharge plus another complication/disease did not differ (*p* = 0.616) between the DIS+/CYTO+ and DIS+/CYTO− cow groups (Figure 3). However, unhealthy cows were more likely to develop CYTO when compared with clinically healthy cows (43.1% (22/51) vs. 20.6% (14/68), *p* = 0.009).

### 3.6. Disease and CYTO Effects on First 45-day Cumulative Milk Yield

**Figure 4:**
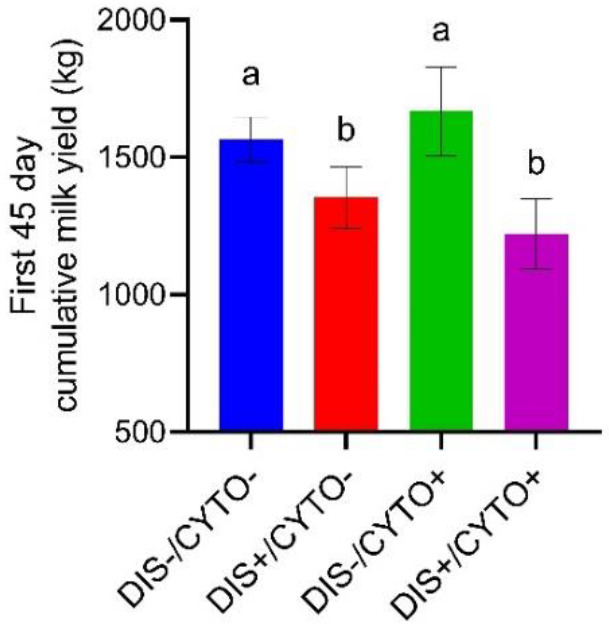
First 45-day cumulative milk yields (means and 95% CI) in 119 multiparous Holstein cows stratified by disease (DIS) and cytological endometritis (CYTO) statuses (DIS−/CYTO− = disease-negative/CYTO-negative; DIS+/CYTO− = disease-positive/CYTO-negative; DIS−/CYTO+ = disease-negative/CYTO-positive; DIS+/CYTO+ = disease-positive/CYTO-positive). Different letters indicate statistical significance at p-values < 0.05.

DIS/CYTO group affected milk yield (*p* < 0.001). The first 45-day cumulative milk yield (mean ± SD) was lower in DIS+/CYTO− (1354.9 ± 286.15 kg) and DIS+/CYTO+ (1221.5 ± 442.13 kg) cows compared with DIS−/CYTO− (1564.9 ± 226.09 kg) and DIS−/CYTO+ (1668.0 ± 341.73 kg) cows. This variable did not differ between the DIS−/CYTO+ and DIS−/CYTO− cow groups (Figure 4).

## 4. Discussion

In this study we demonstrate that the inflammatory status of the endometrium of dairy cows—manifested by an increased proportion of PMN in uterine cytology—can be a disease effect modifier by its capacity to delay onset of luteal activity, reduce pregnancy rates and increase culling risk. Cows that did not have a calving-related condition and/or clinical disease nor a positive CYTO diagnosis had good fertility and a low culling risk. A CYTO-positive status, irrespective of disease status, had a negative effect on fertility. When DIS+ and CYTO+ were combined, they had an additive negative effect. Our data suggest that variation in the CYTO status of examined animals may at least in part explain the heterogeneous disease effects on fertility measures between studies in the literature. Indeed, previous studies investigating the effects of diseases on the reproduction of dairy cows have not accounted for the effects of a positive or negative CYTO status [4,6,9,20,21,22,23].

The terminology used for endometritis in cattle varies widely among researchers [18,36]. We chose to use the term “CYTO” to indicate the proportion of PMN exceeding a threshold associated with decreased pregnancy rates, irrespective of the occurrence of other calving-related disorders and/or clinical diseases. The exclusion of diseased cows from experiments studying the effects of an increased proportion of PMN in the uterine lumen on reproduction explains the lack of data on the combined effects of clinical disease and CYTO on the fertility and culling of dairy cows. Furthermore, unhealthy animals being excluded may have led to an underestimate of the true prevalence of CYTO as well as an underestimate of the effects of CYTO on fertility. The proportions of cows with calving-related conditions and/or clinical diseases (42.9%) and CYTO (30.3%) reported here correspond well with previous reports in the literature [6,12,18,37]. The proportion of cows with clinical uterine inflammatory disease (30.3%) was higher [6], similar [37] or lower [21] than previously reported.

Based on the disease and CYTO status effects on fertility and culling, we distinguished four different phenotypes of cows (Figure 1 and Figure 2), which may reflect immune, metabolic and homeostatic responses and consequently the ability of these animals to adapt to the changes related to parturition and transition to lactation. DIS−/CYTO− cows (45% of the study population) had good fertility and low culling risk, therefore representing a phenotype with adequate adaptation to the transition period. DIS+/CYTO− cows (24% of the study population), despite having calving-related problems and/or clinical diseases, regained homeostasis and did not progress to CYTO. A DIS+/CYTO− health status did not affect the time to onset of luteal activity and did not increase the culling risk. Cows belonging to this phenotype group had superior fertility and lower culling risk compared with DIS−/CYTO+ and DIS+/CYTO+ cows. DIS−/CYTO+ cows (12% of the study population), despite not suffering any calving-related condition/clinical disease, were not able to adapt to parturition and transition to lactation and developed CYTO. DIS−/CYTO+ cows had a decreased pregnancy rate and a trend to increased culling risk; however, DIS−/CYTO+ health status did not affect the time to onset of luteal activity. A DIS+/CYTO+ health status (19% of the study population) mirrored the poorest adaptive ability of the animals to the transition period. In addition to having calving-related disorders and/or clinical diseases, the cows also progressed to CYTO. Cows with a DIS+/CYTO+ health status had the lowest fertility, highest culling risk and resumed postpartum luteal activity later compared with the three other phenotype groups.

Even though cows with clinical health problems early postpartum are medically treated and clinical resolution is generally attained within a few days of treatment, the present data suggest that the far-reaching consequences of clinical diseases in the early postpartum period can be at least partly mediated through the development of CYTO. The biological processes underlying the development of CYTO in dairy cows have not been fully elucidated. CYTO can be a consequence of disease, metabolic stress, energy deficiency and systemic inflammation. The following risk factors associated with the development of CYTO have been reported: assisted calving [10], twins [10], retained placenta [10,38], metritis [12,38], vaginal mucus condition [39], higher concentrations of non-esterified fatty acids one week before [40], at [41] or 35 days after [41] calving, hyper-ketonaemia [12,37], low body condition two weeks before [15], at [37] or after [42] calving, low concentrations of plasma insulin-like growth factor-1 (IGF-1) two weeks before and one week after calving [15], lower blood glucose concentrations at 4 and 6 weeks postpartum [42], lower blood urea nitrogen concentrations at 2 and 4 weeks postpartum [42], elevated serum haptoglobin concentrations during the first week after calving [37], lower concentrations of albumin throughout the transition period [43] and lower concentrations of Mg [43]. Additionally, Moore et al. have reported that the genetic merit for fertility traits affects the proportion of cows with postpartum uterine health based on uterine cytology [44].

In line with previous studies [10,12,38], the proportion of cows that developed CYTO was greater for unhealthy cows compared with healthy cows (43.1 vs. 20.6%, *p* = 0.009). Interestingly, there was no difference in the proportion of cows with uterine and non-uterine diseases between the DIS+/CYTO+ and DIS+/CYTO− cow groups (*p* = 0.616) (Figure 3). Out of all the CYTO-positive cows, obstetrical condition and/or clinical uterine disease did not precede the positive CYTO diagnosis in 61.1% of cases, indicating that obstetrical condition and/or metritis/clinical endometritis was not associated with the development of CYTO in the majority of our cases. Diaz-Lundahl et al. [39] also reported that, in the majority of their CYTO-positive cows (89%), a calving-related problem/obstetrical condition did not precede the positive CYTO diagnosis. Moreover, out of the cows without any calving-related disorders and clinical diseases, we found that 20.6% developed CYTO. This number is in between the prevalence of 11.8% reported by Barlund et al. [45] and that of 34.3% reported by Senosy et al. [42].

Individual cows kept under identical conditions show different adaptations to metabolic stress [46]. The same may apply to postpartum regulation of inflammation [47,48]. Metabolic stress can impair innate immunity in the endometrium [49]. Limiting the availability of glucose or glutamine impairs the ability to mount inflammatory responses to pathogens in endometrial organ cultures [50,51]. It has been suggested that the development of CYTO is a consequence of lack of energy, metabolic stress and systemic inflammation, leading to impaired uterine immune function [12,17,18,19]. Thus, clinically healthy as well as unhealthy cows that progressed to CYTO in our study population may have had a more severe metabolic imbalance, energy deficiency and/or systemic inflammation compared with the cows that did not develop CYTO.

Recent studies strongly suggest that endotoxins play a significant role in the development of multiple periparturient diseases currently affecting transition dairy cows [52,53]. The origin of a higher circulating endotoxin level can be the uterus (metritis/clinical endometritis) [54], mammary glands (mastitis) [55], gastrointestinal tract (subacute ruminal acidosis [56] or an excessively permeable intestine [57]). There is also evidence for a potential role for endotoxins in the aetiology of laminitis [58], left displaced abomasum [53], hypocalcaemia [59], downer cow syndrome [60] and fatty liver disease [52]. Endotoxins activate Kupffer cells which release more proinflammatory cytokines into the systemic circulation [61]. Cytokines, interleukin-1 and interleukin-6 are likely triggers of increased concentrations of acute-phase proteins [62].

Endotoxins are able to disrupt the hypothalamus-pituitary-ovary axis, thus affecting gonadotropin-releasing hormone and preovulatory luteinizing hormone release [63,64], follicle development, preovulatory oestradiol rise [65,66] and ovulation [67]. The endotoxin lipopolysaccharide can alter early embryo-maternal communication by deregulating genes coding for proteins belonging to the galectin family [68]. Concentrations of acute-phase proteins reflect the magnitude of lipopolysaccharide exposure [69]. There is evidence that systemic inflammation is detrimental for early resumption of ovarian cyclicity and an elevated haptoglobin concentration during the postpartum period has been reported to be associated with a longer period of anovulation [70]. Cheong et al. [67] found plasma haptoglobin levels to be significantly higher in non-ovulatory than ovulatory cows on the day of calving and at 3 days postpartum. Moreover, a high haptoglobin concentration 1–7 days postpartum is a risk factor for the development of CYTO [37]. Based on these data, we propose that the DIS+/CYTO+ cows in our study population may have had higher circulating endotoxin and acute-phase protein concentrations, thus explaining why onset of luteal activity was affected (i.e., delayed) in DIS+/CYTO+ cows only (DIS−/CYTO−, DIS−/CYTO+ and DIS+/CYTO− cows had a similar time to onset of luteal activity) (Figure 2). In addition to DIS+/CYTO+ health status, low BCS at week +3 postpartum (≤2.5) was associated with the later start of luteal activity (HR = 0.45, *p* < 0.001). In line with the study of Manríquez et al. [23], there was no statistically significant interaction of disease and CYTO health group by BCS (*p* = 0.750). Manríquez et al. [23] studied combined effect of disease occurrence and the extent of BCS loss from calving until the third week postpartum on subsequent fertility, milk yield and survival and did not find a significant effect of the interaction term ΔBCS by health status for any of the outcomes, including resumption of ovarian cyclicity. Cows that have a low BCS in the early postpartum period are more likely to have negative net energy, which is associated with low luteinizing hormone pulse frequency [71], the availability of insulin and IGF-1 necessary for ovarian follicle stimulation, oestradiol production and ovulation [72,73].

This is the first study to demonstrate the additive effects of clinical disease and CYTO on postpartum onset of luteal activity. Previous studies have presented inconsistent results regarding the effect of CYTO on the interval from calving to first ovulation. In agreement with the findings of Senosy et al. [42], we found that DIS−/CYTO+ health status did not affect the time to onset of luteal activity. A study by Burke et al. [43] reported that the proportions of cows ovulating within either the high or low PMN group were similar through to day 56 postpartum; however, a greater percentage of high PMN cows failed to ovulate before day 63 or 70 compared with low PMN cows (34 and 10%, respectively). Cows with clinical endometritis (Metricheck score > 2) were also included in their study, which may have affected the results. Dourey et al. [74] found that the interval from calving to first ovulation was shorter in low PMN cows than in high PMN cows; however, information on the occurrence of clinical diseases in the studied cows was not provided. As detailed in a study conducted by Dubuc et al. [70], prolonged postpartum anovulation was associated with CYTO. Dubuc et al. [70] purposely selected animals on the basis of having or not having peripartum conditions, which may have affected the results. As the aforementioned studies used different inclusion criteria for the studied cows, this could be the source of variance leading to contradictory results on the effect of CYTO status on resumption of luteal activity.

Both the DIS−/CYTO+ and DIS+/CYTO+ cow groups had reduced fertility and higher culling risk (Figure 1 and Figure 2). An inflammatory milieu in the uterus has been shown to decrease sperm motility, oocyte maturation, corpus luteum function and embryonic quality [75]. PMN infiltration results in an aberrant endometrial transcriptome dynamic between day 0 and day 7 of the oestrous cycle and affects the gene expression profile of bovine preimplantation embryos which may lead to preimplantation embryonic loss [76]. As metabolic stress is considered to be a risk factor for the development of CYTO, the effects of CYTO on fertility could also be in part mediated by mechanisms associated with suboptimal metabolite conditions [77].

The drastically reduced fertility and high culling risk in cows with both clinical disease and CYTO (DIS+/CYTO+ group) may be a combination of effects of compromised follicular growth development, delayed start of ovarian cyclicity, compromised oocyte developmental competence and survival, reduced sperm motility, fertilization failure, low embryo quality and impaired uterine ability to support implantation and pregnancy.

Removal of cows from the herd was largely explained by those that had both clinical disease and CYTO or that were clinically healthy but had CYTO. The odds of removal from the herd were 5.6- and 4.2-fold greater for DIS+/CYTO+ and DIS−/CYTO+ cows, respectively, compared with DIS−/CYTO− cows. Culling was not affected by DIS+/CYTO− health status, indicating that despite DIS+/CYTO− cows having postpartum problems and/or diseases, they were still able to regain homeostasis, resume cyclicity and become pregnant. Getting pregnant explains the similar culling risk for DIS+/CYTO− and DIS−/CYTO− cows. The identification of single and combined effects as potential predictors of impaired reproduction and increased culling of cows is highly beneficial for the goal of improving the management and profitability of dairy herds [78].

An interesting finding was that milk yield during the first 45 days of lactation in DIS−/CYTO+ cows did not differ from DIS−/CYTO− cows (1668 and 1565 kg, respectively, *p* = 1.0). However, DIS−/CYTO+ cows had a lower first AI pregnancy rate than DIS−/CYTO− cows (23.0 and 57.7%, respectively, *p* = 0.034) and were 57% (*p* = 0.019) less likely to become pregnant. Published data on the association of milk production with fertility are conflicting [79]. There are studies which found a negative relationship between milk yield and fertility [80,81,82], while a positive association between high milk yield and fertility has also been reported [79,83]. In the field, dairy cows are not examined for elevated PMN levels in the uterine lumen. Therefore, in studies investigating the relationship between milk yield and fertility, the confounding effect of CYTO has not been accounted for despite its prevalence ranging from 5 to 80% in some herds [12,13,14].

Our data suggest that the CYTO+ health status in clinically healthy high-yielding dairy cows can be an important biological factor influencing fertility outcome, and that at least in part, the variation in fertility in high-yielding dairy cows can be explained by the prevalence of CYTO. Future studies investigating the associations of milk yield and fertility must consider the confounding effect of CYTO on fertility.

## 5. Conclusions

In this study, we demonstrated that CYTO+ status, irrespective of DIS status, decreased the pregnancy rate and increased the time to pregnancy in multiparous cows. DIS+ and CYTO+ health statuses combined had a detrimental effect on the onset of postpartum luteal activity, pregnancy and survival. DIS−/CYTO+ health status (subclinical endometritis) did not affect onset of luteal activity. Our data suggest that the far-reaching consequences of clinical diseases on fertility and culling can be mediated through the development of CYTO. In herds with fertility problems, special attention needs to be paid to the occurrence of CYTO, both in cows with calving-related disorders and/or clinical diseases and in clinically healthy cows.

This study is a proof of concept; future studies with larger number of animals are needed to confirm our findings and to further evaluate the effect of calving-related conditions/clinical diseases and the effect of CYTO on the fertility and culling of cows belonging to different breeds that are kept in different environmental and managemental conditions. Furthermore, the mechanisms underlying why some cows with or without clinical evidence of disease, in similar environmental conditions, develop CYTO and others do not need to be established.

## Figures and Tables

**Table 1:** Descriptive statistics for the 119 studied Holstein cows.

Variable	*n*	Mean ± SD	Range
Parity ^1^	119	2.48 ± 0.75	2–6
First 45-day cumulative milk yield (kg)	119	1462 ± 337	531–2105
Body condition score at week +3 postpartum ^2^	119	2.85 ± 0.31	2–3.75
Interval from calving to onset of luteal activity (days)	117	46.0 ± 27.7	17–147
Calving to first AI interval (days)	112	83.6 ± 15.5	54–136
Pregnancy rate after first AI (%)	48/112	42.9	
Culling rate (%)	19/119	16.0	

^1^ Of the cows, 64% (*n* = 76) were 2nd lactation and 36% (*n* = 43) were >2nd lactation. ^2^ Of the cows, 21% (*n* = 25) had body condition score (BCS) ≤ 2.5 and 79% (*n* = 94) had BCS ≥ 2.75. AI: artificial insemination.

## Data Availability

The data that support the findings of this study are available on reasonable request from the senior author.
